# *catena*-Poly[[(1,10-phenanthroline)lead(II)]bis­(μ-5-chloro-2-hy­droxy­benzoato)]

**DOI:** 10.1107/S1600536810023561

**Published:** 2010-07-21

**Authors:** Lei Yang, Bing Li, Qing Xue, Yu Huo, Gaopeng Wang

**Affiliations:** a Department of Physics–Chemistry, Henan Polytechnic University, Jiao Zuo, 454150, People’s Republic of China; b Department of Chemistry, Zhejiang Forestry University, Lin’an, 311300, People’s Republic of China

## Abstract

In the title polymer, [Pb(C_7_H_4_ClO_3_)_2_(C_12_H_8_N_2_)]_*n*_, the Pb(II) ion displays a distorted pseudo-octa­hedral coordination geometry. The metal center is coordinated by six O atoms from four 5-chloro­salicylate ligands and two N atoms from a chelating phenanthroline ligand. The polymeric structure is built up from bridging carboxyl­ate O atoms, forming chains along [100]. The crystal structure is stabilized by π–π inter­actions between the 1,10-phenanthroline and 5-chloro­salicylate ligands, the shortest centroid–centroid separation between neighbouring aromatic rings being 3.652 (1) Å.

## Related literature

For related non-polymeric complexes including 5-chloro­salicylate ligands, see: Wen & Ying (2007 ▶); Wen *et al.* (2008 ▶).
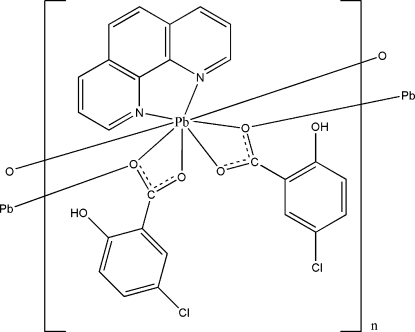


## Experimental

### 

#### Crystal data

[Pb(C_7_H_4_ClO_3_)_2_(C_12_H_8_N_2_)]
*M* *_r_* = 730.51
Triclinic, 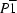

*a* = 8.9100 (1) Å
*b* = 11.2959 (1) Å
*c* = 13.5816 (1) Å
α = 75.508 (1)°
β = 86.302 (1)°
γ = 68.342 (1)°
*V* = 1229.43 (2) Å^3^
*Z* = 2
Mo *K*α radiation
μ = 7.13 mm^−1^
*T* = 296 K
0.28 × 0.25 × 0.24 mm

#### Data collection

Bruker APEXII CCD diffractometer
Absorption correction: multi-scan (*SADABS*; Bruker, 2001 ▶) *T*_min_ = 0.156, *T*_max_ = 0.181
21579 measured reflections
5987 independent reflections
5339 reflections with *I* > 2σ(*I*)
*R*_int_ = 0.029

#### Refinement

*R*[*F* ^2^ > 2σ(*F* ^2^)] = 0.025
*wR*(*F* ^2^) = 0.064
*S* = 1.05
5987 reflections
334 parameters
H-atom parameters constrained
Δρ_max_ = 1.18 e Å^−3^
Δρ_min_ = −0.59 e Å^−3^

### 

Data collection: *APEX2* (Bruker, 2001 ▶); cell refinement: *SAINT* (Bruker, 2001 ▶); data reduction: *SAINT*; program(s) used to solve structure: *SHELXS97* (Sheldrick, 2008 ▶); program(s) used to refine structure: *SHELXL97* (Sheldrick, 2008 ▶); molecular graphics: *SHELXTL* (Sheldrick, 2008 ▶); software used to prepare material for publication: *SHELXTL*.

## Supplementary Material

Crystal structure: contains datablocks I, global. DOI: 10.1107/S1600536810023561/bh2289sup1.cif

Structure factors: contains datablocks I. DOI: 10.1107/S1600536810023561/bh2289Isup2.hkl

Additional supplementary materials:  crystallographic information; 3D view; checkCIF report

## Figures and Tables

**Fig. 1.**
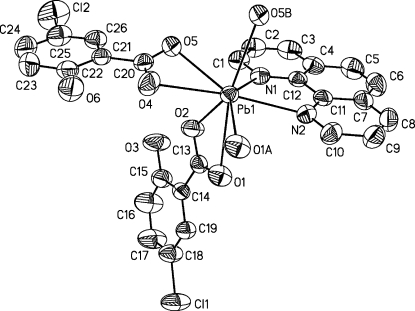
The Pb coordination geometry in the title compound, with thermal ellipsoids at the 50% probability level. Symmetry codes: A 1 - x, -y, -z; B: -x, -y, -z.

**Fig. 2.**
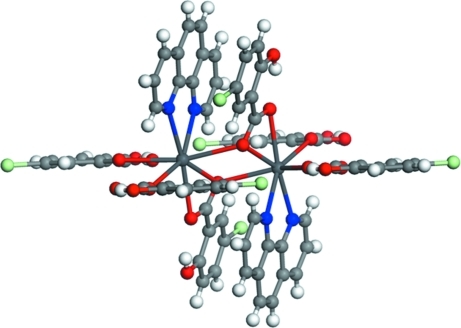
A section of the structure of the title complex showing SBU with shared corner O atoms.

**Fig. 3.**
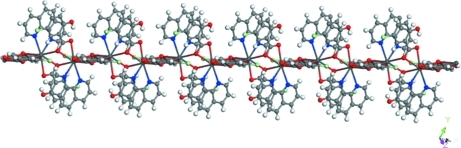
The chain of the title polymer, viewed down the a axis.

## supplementary crystallographic information

### Comment

The Pb(II) ion in the asymmetric unit is pseudo-octahedrally coordinated (Fig. 1), with coordination to six O atoms from four 5-chlorosalicylate ligands, and two N atoms from a chelating phenanthroline (phen) ligand. Two related complexes, [Zn(C_7_H_4_ClO_3_)_2_(C_12_H_8_N_2_)(H_2_O)] and [Cd(C_7_H_4_ClO_3_)_2_(C_12_H_8_N_2_)_2_], were reported (Wen & Ying, 2007; Wen *et al.*, 2008, respectively), both with 5-chlorosalicylate ions acting as monodentate ligands, while in the title polymer, 5-chlorosalicylate ions are bidentate ligands.

The Pb—O distances (Table 1) vary from 2.458 (3) to 2.887 (3) Å, which are close to those found in Pb_2_(*PMIDA*).1.5H_2_O [from 2.331 (9) to 2.876 (9) Å; H_4_*PMIDA* is *N*-(phosphonomethyl)iminodiacetic acid]. Each pair of adjacent Pb(II) ions is bridged by the O1 atom of 5-chlorosalicylate, which forms a SBU (secondary building units, Fig. 2) including two Pb polyhedron, six 5-chlorosalicylate and two phen. The Pb···Pb distance in the dimeric unit is 4.3587 (2) Å. The SBU is bridged by O5 atom of 5-chlorosalicylate, to give rise to a zigzag chain (Fig. 3). The excellent coordinating ability and large conjugated systems of phen and 5-chlorosalicylate allow to form π···π interactions. The chains are extended into the framework through π..π interactions between the ligands from adjacent chains.

### Experimental

The pH value of a mixture of Pb(NO_3_)_2_ (0.5 mmol), phen (0.5 mmol) and 5-chlorosalicylic acid (0.5 mmol) in 8 ml of distilled water and 16 ml of ethanol was adjusted between 5 and 6 by addition of sodium hydroxide. The resultant solution was refluxed for 6 h. After cooling, yellow crystals were formed over 2 days, at room temperature.

### Refinement

H atoms were placed geometrically with bond lengths fixed to 0.82 (OH) and 0.93 Å (CH). Isotropic displacement parameters for H atoms were calculated as *U*_iso_(H) = 1.2*U*_eq_(carrier C) and *U*_iso_(H) = 1.5*U*_eq_(carrier O).

### Crystal data

**Table:**

[Pb(C_7_H_4_ClO_3_)_2_(C_12_H_8_N_2_)]	*Z* = 2
*M**_r_* = 730.51	*F*(000) = 700
Triclinic, *P*1	*D*_x_ = 1.973 Mg m^−^^3^
Hall symbol: -P 1	Mo *K*α radiation, λ = 0.71073 Å
*a* = 8.9100 (1) Å	Cell parameters from 5987 reflections
*b* = 11.2959 (1) Å	θ = 2.8–26.9°
*c* = 13.5816 (1) Å	µ = 7.13 mm^−^^1^
α = 75.508 (1)°	*T* = 296 K
β = 86.302 (1)°	Block, yellow
γ = 68.342 (1)°	0.28 × 0.25 × 0.24 mm
*V* = 1229.43 (2) Å^3^	

### Data collection

**Table:**

Bruker APEXII CCD diffractometer	5987 independent reflections
Radiation source: fine-focus sealed tube	5339 reflections with *I* > 2σ(*I*)
graphite	*R*_int_ = 0.029
φ and ω scans	θ_max_ = 28.2°, θ_min_ = 2.0°
Absorption correction: multi-scan (*SADABS*; Bruker, 2001)	*h* = −11→11
*T*_min_ = 0.156, *T*_max_ = 0.181	*k* = −14→14
21579 measured reflections	*l* = −18→17

### Refinement

**Table:**

Refinement on *F*^2^	Primary atom site location: structure-invariant direct methods
Least-squares matrix: full	Secondary atom site location: difference Fourier map
*R*[*F*^2^ > 2σ(*F*^2^)] = 0.025	Hydrogen site location: inferred from neighbouring sites
*wR*(*F*^2^) = 0.064	H-atom parameters constrained
*S* = 1.05	*w* = 1/[σ^2^(*F*_o_^2^) + (0.0349*P*)^2^ + 0.472*P*] where *P* = (*F*_o_^2^ + 2*F*_c_^2^)/3
5987 reflections	(Δ/σ)_max_ = 0.001
334 parameters	Δρ_max_ = 1.18 e Å^−^^3^
0 restraints	Δρ_min_ = −0.59 e Å^−^^3^
0 constraints	

### Fractional atomic coordinates and isotropic or equivalent isotropic displacement parameters (Å^2^)

**Table:**

	*x*	*y*	*z*	*U*_iso_*/*U*_eq_	
Pb1	0.258486 (14)	−0.038882 (12)	−0.001073 (10)	0.03675 (5)	
Cl1	1.04796 (14)	−0.31797 (16)	0.36720 (10)	0.0779 (4)	
Cl2	−0.2949 (2)	−0.0117 (2)	0.47404 (14)	0.1106 (6)	
O5	0.0025 (3)	0.0001 (3)	0.1216 (2)	0.0495 (6)	
O1	0.5607 (4)	−0.1240 (3)	0.0939 (2)	0.0548 (7)	
O2	0.3533 (3)	−0.1567 (3)	0.1750 (2)	0.0569 (7)	
O4	0.1160 (4)	0.1481 (3)	0.1053 (2)	0.0604 (8)	
N1	0.2350 (4)	−0.2615 (3)	0.0256 (2)	0.0423 (7)	
N2	0.4307 (4)	−0.1896 (3)	−0.1218 (3)	0.0449 (7)	
C12	0.2979 (4)	−0.3371 (3)	−0.0407 (3)	0.0394 (8)	
O3	0.3774 (4)	−0.2835 (5)	0.3602 (3)	0.0882 (13)	
H3B	0.3326	−0.2436	0.3043	0.132*	
C11	0.3981 (4)	−0.2980 (4)	−0.1197 (3)	0.0409 (8)	
C13	0.5001 (4)	−0.1665 (3)	0.1727 (3)	0.0417 (8)	
C14	0.5973 (4)	−0.2334 (4)	0.2701 (3)	0.0421 (8)	
C15	0.5322 (5)	−0.2888 (6)	0.3575 (3)	0.0609 (12)	
C7	0.4610 (5)	−0.3747 (4)	−0.1912 (3)	0.0509 (10)	
C8	0.5574 (6)	−0.3329 (5)	−0.2661 (4)	0.0615 (12)	
H8A	0.6005	−0.3804	−0.3148	0.074*	
O6	0.0401 (4)	0.3062 (3)	0.2185 (3)	0.0704 (9)	
H6B	0.0809	0.2756	0.1702	0.106*	
C21	−0.0455 (4)	0.1217 (4)	0.2486 (3)	0.0419 (8)	
C4	0.2689 (5)	−0.4538 (4)	−0.0344 (4)	0.0496 (9)	
C1	0.1447 (5)	−0.2992 (4)	0.0995 (3)	0.0506 (9)	
H1A	0.1000	−0.2465	0.1446	0.061*	
C22	−0.0349 (5)	0.2293 (5)	0.2767 (3)	0.0512 (10)	
C19	0.7579 (5)	−0.2452 (4)	0.2740 (3)	0.0449 (8)	
H19A	0.8040	−0.2107	0.2160	0.054*	
C20	0.0289 (4)	0.0873 (4)	0.1522 (3)	0.0447 (9)	
C9	0.5890 (6)	−0.2234 (5)	−0.2688 (4)	0.0634 (12)	
H9A	0.6523	−0.1946	−0.3191	0.076*	
C2	0.1143 (6)	−0.4148 (5)	0.1118 (4)	0.0590 (11)	
H2A	0.0533	−0.4396	0.1655	0.071*	
C10	0.5239 (5)	−0.1554 (5)	−0.1941 (3)	0.0551 (10)	
H10A	0.5475	−0.0814	−0.1952	0.066*	
C26	−0.1257 (5)	0.0475 (4)	0.3101 (3)	0.0510 (9)	
H26A	−0.1347	−0.0236	0.2916	0.061*	
C3	0.1744 (6)	−0.4899 (4)	0.0450 (4)	0.0616 (12)	
H3A	0.1529	−0.5660	0.0517	0.074*	
C5	0.3351 (6)	−0.5275 (5)	−0.1086 (4)	0.0664 (13)	
H5A	0.3147	−0.6033	−0.1054	0.080*	
C6	0.4259 (6)	−0.4894 (5)	−0.1825 (4)	0.0660 (13)	
H6A	0.4673	−0.5396	−0.2297	0.079*	
C25	−0.1914 (6)	0.0793 (5)	0.3981 (3)	0.0638 (12)	
C24	−0.1821 (6)	0.1881 (6)	0.4256 (4)	0.0740 (15)	
H24A	−0.2285	0.2099	0.4851	0.089*	
C23	−0.1051 (6)	0.2610 (6)	0.3650 (4)	0.0688 (13)	
H23A	−0.0993	0.3335	0.3830	0.083*	
C18	0.8483 (5)	−0.3069 (5)	0.3622 (3)	0.0616 (12)	
C17	0.7827 (7)	−0.3620 (7)	0.4485 (4)	0.0867 (19)	
H17A	0.8447	−0.4035	0.5085	0.104*	
C16	0.6277 (7)	−0.3553 (7)	0.4453 (4)	0.090 (2)	
H16A	0.5853	−0.3956	0.5025	0.108*	

### Atomic displacement parameters (Å^2^)

**Table:**

	*U*^11^	*U*^22^	*U*^33^	*U*^12^	*U*^13^	*U*^23^
Pb1	0.03379 (8)	0.04199 (8)	0.03692 (8)	−0.01778 (5)	0.00344 (5)	−0.00846 (5)
Cl1	0.0494 (6)	0.1113 (10)	0.0641 (8)	−0.0373 (7)	−0.0139 (5)	0.0104 (7)
Cl2	0.1064 (12)	0.1390 (15)	0.0809 (10)	−0.0634 (11)	0.0396 (9)	0.0020 (10)
O5	0.0489 (15)	0.0541 (15)	0.0449 (16)	−0.0154 (13)	−0.0012 (12)	−0.0156 (13)
O1	0.0612 (18)	0.0596 (17)	0.0402 (16)	−0.0272 (15)	−0.0072 (13)	0.0037 (13)
O2	0.0420 (15)	0.0743 (19)	0.0543 (18)	−0.0233 (14)	−0.0065 (13)	−0.0106 (15)
O4	0.0583 (18)	0.072 (2)	0.0588 (19)	−0.0311 (16)	0.0224 (15)	−0.0228 (16)
N1	0.0386 (16)	0.0421 (15)	0.0465 (18)	−0.0169 (13)	0.0001 (13)	−0.0076 (13)
N2	0.0396 (16)	0.0503 (18)	0.0479 (19)	−0.0189 (14)	0.0051 (14)	−0.0144 (15)
C12	0.0337 (17)	0.0355 (17)	0.044 (2)	−0.0068 (14)	−0.0094 (15)	−0.0072 (14)
O3	0.054 (2)	0.168 (4)	0.055 (2)	−0.063 (2)	0.0100 (16)	−0.016 (2)
C11	0.0334 (17)	0.0426 (18)	0.043 (2)	−0.0090 (15)	−0.0082 (15)	−0.0089 (15)
C13	0.0431 (19)	0.0386 (18)	0.046 (2)	−0.0132 (15)	−0.0020 (16)	−0.0157 (16)
C14	0.0409 (19)	0.051 (2)	0.038 (2)	−0.0194 (16)	0.0004 (15)	−0.0123 (16)
C15	0.052 (2)	0.097 (4)	0.042 (2)	−0.037 (2)	0.0066 (19)	−0.017 (2)
C7	0.047 (2)	0.049 (2)	0.048 (2)	−0.0041 (17)	−0.0109 (18)	−0.0144 (18)
C8	0.059 (3)	0.073 (3)	0.045 (2)	−0.010 (2)	0.003 (2)	−0.023 (2)
O6	0.068 (2)	0.075 (2)	0.085 (3)	−0.0421 (18)	0.0217 (18)	−0.0284 (19)
C21	0.0324 (17)	0.054 (2)	0.0364 (19)	−0.0137 (16)	−0.0002 (14)	−0.0095 (16)
C4	0.050 (2)	0.0374 (19)	0.058 (2)	−0.0137 (17)	−0.0157 (19)	−0.0045 (17)
C1	0.050 (2)	0.051 (2)	0.050 (2)	−0.0226 (18)	0.0022 (18)	−0.0045 (18)
C22	0.038 (2)	0.065 (3)	0.052 (2)	−0.0196 (19)	0.0005 (17)	−0.016 (2)
C19	0.0416 (19)	0.052 (2)	0.039 (2)	−0.0193 (17)	0.0012 (16)	−0.0035 (16)
C20	0.0338 (18)	0.055 (2)	0.040 (2)	−0.0095 (16)	−0.0021 (15)	−0.0103 (17)
C9	0.053 (3)	0.079 (3)	0.055 (3)	−0.020 (2)	0.010 (2)	−0.017 (2)
C2	0.054 (2)	0.058 (3)	0.061 (3)	−0.029 (2)	0.000 (2)	0.005 (2)
C10	0.050 (2)	0.064 (3)	0.056 (3)	−0.026 (2)	0.014 (2)	−0.018 (2)
C26	0.044 (2)	0.061 (2)	0.046 (2)	−0.0218 (19)	0.0014 (17)	−0.0048 (18)
C3	0.061 (3)	0.046 (2)	0.078 (3)	−0.027 (2)	−0.011 (2)	−0.003 (2)
C5	0.072 (3)	0.045 (2)	0.085 (4)	−0.019 (2)	−0.014 (3)	−0.021 (2)
C6	0.073 (3)	0.054 (2)	0.069 (3)	−0.010 (2)	−0.007 (3)	−0.029 (2)
C25	0.052 (2)	0.088 (3)	0.043 (2)	−0.028 (2)	0.0101 (19)	0.000 (2)
C24	0.061 (3)	0.111 (4)	0.047 (3)	−0.022 (3)	0.012 (2)	−0.030 (3)
C23	0.065 (3)	0.087 (3)	0.063 (3)	−0.028 (3)	0.011 (2)	−0.036 (3)
C18	0.049 (2)	0.090 (3)	0.044 (2)	−0.033 (2)	−0.0051 (19)	−0.001 (2)
C17	0.068 (3)	0.146 (6)	0.042 (3)	−0.053 (4)	−0.011 (2)	0.010 (3)
C16	0.078 (4)	0.157 (6)	0.040 (3)	−0.067 (4)	0.001 (2)	0.006 (3)

### Geometric parameters (Å, °)

**Table:**

Pb1—O2	2.458 (3)	C8—H8A	0.9300
Pb1—O5	2.703 (3)	O6—C22	1.358 (6)
Pb1—O4	2.734 (3)	O6—H6B	0.8200
Pb1—O5^i^	2.768 (3)	C21—C26	1.388 (6)
Pb1—O1	2.785 (3)	C21—C22	1.399 (6)
Pb1—O1^ii^	2.887 (3)	C21—C20	1.502 (5)
Pb1—N1	2.534 (3)	C4—C3	1.398 (7)
Pb1—N2	2.662 (3)	C4—C5	1.426 (7)
Cl1—C18	1.742 (4)	C1—C2	1.397 (6)
Cl2—C25	1.737 (5)	C1—H1A	0.9300
O5—C20	1.259 (5)	C22—C23	1.382 (6)
O5—Pb1^i^	2.768 (3)	C19—C18	1.364 (6)
O1—C13	1.235 (5)	C19—H19A	0.9300
O1—Pb1^ii^	2.887 (3)	C9—C10	1.393 (7)
O2—C13	1.270 (5)	C9—H9A	0.9300
O4—C20	1.261 (5)	C2—C3	1.348 (7)
N1—C1	1.331 (5)	C2—H2A	0.9300
N1—C12	1.350 (5)	C10—H10A	0.9300
N2—C10	1.321 (5)	C26—C25	1.366 (6)
N2—C11	1.353 (5)	C26—H26A	0.9300
C12—C4	1.416 (5)	C3—H3A	0.9300
C12—C11	1.439 (5)	C5—C6	1.336 (8)
O3—C15	1.357 (5)	C5—H5A	0.9300
O3—H3B	0.8200	C6—H6A	0.9300
C11—C7	1.415 (5)	C25—C24	1.402 (8)
C13—C14	1.495 (5)	C24—C23	1.354 (8)
C14—C19	1.390 (5)	C24—H24A	0.9300
C14—C15	1.395 (6)	C23—H23A	0.9300
C15—C16	1.386 (7)	C18—C17	1.385 (7)
C7—C8	1.394 (7)	C17—C16	1.358 (7)
C7—C6	1.417 (7)	C17—H17A	0.9300
C8—C9	1.358 (7)	C16—H16A	0.9300
			
O2—Pb1—N1	74.00 (10)	O2—C13—C14	116.7 (4)
O2—Pb1—N2	107.51 (10)	O1—C13—Pb1	68.5 (2)
N1—Pb1—N2	63.52 (10)	O2—C13—Pb1	53.5 (2)
O2—Pb1—O5	70.89 (9)	C14—C13—Pb1	170.2 (3)
N1—Pb1—O5	81.85 (9)	C19—C14—C15	118.5 (4)
N2—Pb1—O5	143.50 (9)	C19—C14—C13	120.0 (3)
O2—Pb1—O4	76.94 (10)	C15—C14—C13	121.5 (3)
N1—Pb1—O4	128.10 (10)	O3—C15—C16	117.5 (4)
N2—Pb1—O4	168.25 (9)	O3—C15—C14	122.5 (4)
O5—Pb1—O4	48.05 (9)	C16—C15—C14	120.0 (4)
O2—Pb1—O5^i^	135.16 (9)	C8—C7—C11	117.1 (4)
N1—Pb1—O5^i^	73.83 (9)	C8—C7—C6	123.7 (4)
N2—Pb1—O5^i^	84.71 (9)	C11—C7—C6	119.2 (4)
O5—Pb1—O5^i^	74.36 (9)	C9—C8—C7	120.6 (4)
O4—Pb1—O5^i^	99.85 (9)	C9—C8—H8A	119.7
O2—Pb1—O1	48.91 (9)	C7—C8—H8A	119.7
N1—Pb1—O1	97.78 (9)	C22—O6—H6B	109.5
N2—Pb1—O1	81.42 (10)	C26—C21—C22	119.5 (4)
O5—Pb1—O1	116.23 (9)	C26—C21—C20	120.4 (4)
O4—Pb1—O1	94.20 (10)	C22—C21—C20	120.1 (4)
O5^i^—Pb1—O1	165.95 (9)	C3—C4—C12	117.3 (4)
O2—Pb1—O1^ii^	111.66 (9)	C3—C4—C5	123.5 (4)
N1—Pb1—O1^ii^	145.69 (10)	C12—C4—C5	119.2 (4)
N2—Pb1—O1^ii^	83.09 (9)	N1—C1—C2	122.6 (4)
O5—Pb1—O1^ii^	132.42 (9)	N1—C1—H1A	118.7
O4—Pb1—O1^ii^	85.17 (9)	C2—C1—H1A	118.7
O5^i^—Pb1—O1^ii^	112.58 (8)	O6—C22—C23	118.4 (4)
O1—Pb1—O1^ii^	68.01 (9)	O6—C22—C21	121.9 (4)
O2—Pb1—C13	24.55 (10)	C23—C22—C21	119.7 (4)
N1—Pb1—C13	85.87 (10)	C18—C19—C14	120.6 (4)
N2—Pb1—C13	94.97 (10)	C18—C19—H19A	119.7
O5—Pb1—C13	93.59 (10)	C14—C19—H19A	119.7
O4—Pb1—C13	84.95 (10)	O5—C20—O4	122.8 (4)
O5^i^—Pb1—C13	157.51 (10)	O5—C20—C21	119.4 (4)
O1—Pb1—C13	24.36 (9)	O4—C20—C21	117.8 (4)
O1^ii^—Pb1—C13	89.63 (10)	O5—C20—Pb1	62.0 (2)
O2—Pb1—C20	68.44 (10)	O4—C20—Pb1	63.4 (2)
N1—Pb1—C20	103.98 (11)	C21—C20—Pb1	163.5 (2)
N2—Pb1—C20	167.39 (11)	C8—C9—C10	118.2 (5)
O5—Pb1—C20	24.29 (10)	C8—C9—H9A	120.9
O4—Pb1—C20	24.36 (10)	C10—C9—H9A	120.9
O5^i^—Pb1—C20	90.21 (9)	C3—C2—C1	119.2 (4)
O1—Pb1—C20	102.90 (9)	C3—C2—H2A	120.4
O1^ii^—Pb1—C20	109.53 (11)	C1—C2—H2A	120.4
C13—Pb1—C20	85.33 (10)	N2—C10—C9	124.0 (5)
O2—Pb1—Pb1^i^	104.32 (7)	N2—C10—H10A	118.0
N1—Pb1—Pb1^i^	74.65 (7)	C9—C10—H10A	118.0
N2—Pb1—Pb1^i^	116.10 (7)	C25—C26—C21	119.7 (4)
O5—Pb1—Pb1^i^	37.70 (6)	C25—C26—H26A	120.1
O4—Pb1—Pb1^i^	72.18 (7)	C21—C26—H26A	120.1
O5^i^—Pb1—Pb1^i^	36.66 (6)	C2—C3—C4	120.3 (4)
O1—Pb1—Pb1^i^	152.88 (6)	C2—C3—H3A	119.8
O1^ii^—Pb1—Pb1^i^	131.46 (6)	C4—C3—H3A	119.8
C13—Pb1—Pb1^i^	128.72 (8)	C6—C5—C4	121.2 (4)
C20—Pb1—Pb1^i^	55.75 (7)	C6—C5—H5A	119.4
C20—O5—Pb1	93.7 (2)	C4—C5—H5A	119.4
C20—O5—Pb1^i^	126.0 (2)	C5—C6—C7	121.9 (4)
Pb1—O5—Pb1^i^	105.64 (9)	C5—C6—H6A	119.0
C13—O1—Pb1	87.2 (2)	C7—C6—H6A	119.0
C13—O1—Pb1^ii^	145.9 (3)	C26—C25—C24	120.7 (4)
Pb1—O1—Pb1^ii^	111.99 (9)	C26—C25—Cl2	119.8 (4)
C13—O2—Pb1	101.9 (3)	C24—C25—Cl2	119.5 (4)
C20—O4—Pb1	92.2 (2)	C23—C24—C25	119.5 (4)
C1—N1—C12	118.6 (3)	C23—C24—H24A	120.3
C1—N1—Pb1	119.9 (3)	C25—C24—H24A	120.3
C12—N1—Pb1	121.1 (2)	C24—C23—C22	120.9 (5)
C10—N2—C11	117.9 (4)	C24—C23—H23A	119.6
C10—N2—Pb1	124.7 (3)	C22—C23—H23A	119.6
C11—N2—Pb1	116.5 (2)	C19—C18—C17	120.4 (4)
N1—C12—C4	121.9 (4)	C19—C18—Cl1	120.3 (3)
N1—C12—C11	118.9 (3)	C17—C18—Cl1	119.3 (4)
C4—C12—C11	119.2 (4)	C16—C17—C18	119.9 (5)
C15—O3—H3B	109.5	C16—C17—H17A	120.1
N2—C11—C7	122.2 (4)	C18—C17—H17A	120.1
N2—C11—C12	118.5 (3)	C17—C16—C15	120.5 (5)
C7—C11—C12	119.3 (4)	C17—C16—H16A	119.8
O1—C13—O2	122.0 (4)	C15—C16—H16A	119.8
O1—C13—C14	121.3 (3)		

Symmetry codes: (i) −*x*, −*y*, −*z*; (ii) −*x*+1, −*y*, −*z*.
